# Postharvest Quality Deterioration Mechanisms and Preservation Technologies of Tea (*Camellia sinensis*): A Review

**DOI:** 10.3390/foods15203766

**Published:** 2026-10-21

**Authors:** Xiangyang Guo

**Affiliations:** College of Tea and Food Science, Xinyang Normal University, No. 237 Nanhu Road, Xinyang 464000, China; xiangyang.guo@ahau.edu.cn

**Keywords:** tea (*Camellia sinensis* (L.) O. Kuntze), postharvest storage, preservation technology, cold chain, modified atmosphere packaging, aroma stability

## Abstract

Tea, a globally important cash crop with abundant bioactive constituents, suffers widespread postharvest quality deterioration that causes substantial economic losses. This review compares the divergence of deterioration pathways between postharvest fresh tea leaves and six major processed tea categories, distinguishes undesired deterioration from desirable maturation in aging-oriented categories such as Pu-erh and aged oolong, and integrates universal core mechanisms with category-specific regulatory characteristics. It identifies ascorbic acid and chlorophyll as reliable freshness indicators for green tea, evaluates mainstream preservation technologies across industrial-adaptability dimensions including cost, scalability, and technical maturity, and analyzes the coupled regulatory effects of moisture, oxygen, temperature, and light. A three-tier whole-chain staged precise preservation framework is proposed, covering source-end fresh-leaf cold-chain transportation, processed-tea storage, and terminal open-state distribution, an integration not explicitly structured in prior tea-storage reviews. The review further discusses industrial challenges and outlines directions for green, precise, composite, intelligent, and standardized preservation, offering systematic references for whole-chain tea quality regulation and supporting the industrial translation of low-carbon technologies.

## 1. Introduction

Tea ranks among the three major non-alcoholic beverages consumed globally. Renowned for its distinct flavor profile, abundant bioactive constituents, and profound cultural connotations, tea has emerged as one of the most widely consumed health-promoting commodities worldwide [1]. In recent years, the upgrading of health-conscious consumption and the expansion of cross-regional tea trade have complicated the full-chain circulation from production to household storage, requiring more precise control of the postharvest quality stability of tea.

As a highly bioactive agricultural product prone to deterioration, tea quality depends heavily on precise regulation of endogenous metabolism. Processed tea retains abundant heat-labile and oxidizable components such as polyphenols, amino acids and chlorophyll due to incomplete enzyme inactivation during manufacturing, driving continuous enzymatic and non-enzymatic reactions throughout storage [2]. Under the synergistic effects of temperature, humidity, oxygen and light, tea suffers from quality degradation—manifesting as pigment browning, polyphenol oxidation and lipid rancidity—that is largely irreversible for unfermented and lightly fermented teas, resulting in turbid infusion, weakened taste and aroma loss [3]. Excessive humidity further promotes microbial proliferation and potential aflatoxin production, threatening drinking safety [4]. Such deterioration causes substantial economic losses and acts as a core bottleneck restricting the high-quality and international development of the tea industry.

Notably, full-chain quality control of tea covers the whole process from postharvest fresh leaves to finished product storage, among which the long-distance transportation of fresh leaves is the most overlooked upstream link. The fresh-leaf storage and transportation examined here concerns pre-processing preservation aimed at maintaining leaf freshness before the withering stage, rather than the intentional withering process that induces controlled water loss and enzymatic transformation to develop aroma precursors. Early studies focused mostly on component changes in processed tea during storage, with limited understanding of the molecular mechanisms underlying postharvest senescence and metabolism in fresh leaves, which has left fresh-leaf storage and transportation technologies at an empirical level for a long time. Although multi-omics technologies have enabled molecular-level deciphering of deterioration regulatory networks in fresh leaves [2], core issues such as systematic differences in deterioration mechanisms between fresh leaves and processed tea remain unresolved.

Tea production and distribution feature seasonal concentration and regional agglomeration. Premium tea is harvested mainly in spring, while consumer demand lasts all year round, making traditional extensive storage modes prone to severe quality loss. Mature preservation technologies can delay tea aging, extend shelf life, stabilize batch quality and address cross-seasonal and cross-regional circulation challenges, while also supporting compliance with international food safety standards. Diverse scenarios from enterprise warehousing to household storage all demand safe, stable and cost-effective preservation technologies, and traditional simple storage methods can no longer meet the upgrading consumption demand.

Despite recent progress in tea storage and preservation research, key challenges remain. The differential deterioration mechanisms among tea categories and between fresh leaves and processed tea have not been fully clarified. Studies on environmental factor coupling effects and targeted aroma retention technologies are still insufficient, and the industrialization of green preservation technologies is progressing slowly. Several prior reviews have addressed aspects of tea storage. Gao et al. [5] provided a comprehensive overview of chemical composition changes and storage technologies but did not differentiate fresh-leaf postharvest metabolism from processed-tea deterioration. Li et al. [4] focused specifically on green-tea storage and preservation and left cross-category comparison unaddressed. Lv et al. [3] broadly examined tea storage, storage-duration identification, pre-storage interventions, and future precision storage but did not construct an integrated whole-chain framework that links source-end fresh-leaf transportation, processed-tea storage, and terminal distribution. This has resulted in a disconnect between laboratory research and industrial standardized application.

Building on existing research, this review compares how deterioration mechanisms differ between postharvest fresh tea leaves and the six major processed tea categories and identifies the environmental factors governing these differences. It evaluates how mainstream preservation technologies compare in efficacy, limitations, and industrial adaptability across tea categories and proposes an integrated whole-chain staged framework to guide precise preservation from source to terminal distribution, addressing the gap of targeted regulation systems. The review further dissects current industrial challenges and outlines future development directions, aiming to provide theoretical references for full-chain quality regulation and the high-quality development of the tea industry.

## 2. Mechanisms Underlying Postharvest Quality Deterioration of Tea

Postharvest quality changes in tea represent a dynamic process driven by the synergistic interplay between physical alterations and biochemical reactions, spanning the entire industrial chain from freshly harvested leaves to end-point consumption. For unfermented and lightly fermented teas, this process is largely irreversible and manifests as quality degradation, whereas for post-fermented teas and certain aged oolongs and white teas, controlled storage can induce desirable maturation that improves sensory quality. For freshness-oriented categories, this degradative process constitutes the fundamental cause of tea aging and underpins the theoretical framework for the development of preservation technologies, process optimization, and industrial implementation. Marked variations in deterioration profiles exist across distinct tea categories. Premium green tea exhibits high sensitivity to ambient environmental conditions and is susceptible to leaf yellowing, infusion darkening, aroma attenuation, and taste dilution during storage and transportation. Black tea deterioration primarily manifests as oxidative browning of pigment compounds, which readily causes darkening of the leaf base and diminished briskness of the tea infusion.

From a metabolic perspective, exposure to storage environments triggers oxidation, degradation, and structural transformation of key endogenous constituents, including tea polyphenols, amino acids, chlorophyll, and lipids. These reactions generate an array of adverse secondary metabolites, which ultimately drive comprehensive quality degradation across color, aroma, taste, and appearance attributes. Metabolic patterns of detached fresh leaves and processed tea exhibit consistent trends under high-temperature stress, with elevated temperatures significantly exacerbating the aberrant transformation of constituents including polyphenols and amino acids [2]. Under conventional commercial storage regimes, phenolic and flavonoid contents and corresponding free radical scavenging activities in green tea, black tea, white tea, and oolong tea all decline progressively with extended storage duration [6]. Furthermore, high-humidity environments further exacerbate reduction in the antioxidant capacity of black tea and green tea [7]. A systematic comparison of postharvest deterioration characteristics across fresh tea leaves and six major tea categories is summarized in Table 1.

### 2.1. Deterioration Profiles and Underlying Mechanisms of Postharvest Fresh Tea Leaves

Following detachment from the tea plant, fresh leaves sustain active respiratory and transpiration processes. Field heat and metabolic heat accumulate readily within leaf piles, inducing a sharp reduction in the physical stability of the leaf tissue. During long-distance transportation under ambient-temperature conditions, fresh leaves are susceptible to physical damage including water loss, wilting, and increased leaf brittleness. Concurrently, water vapor and exogenous odors from the surrounding environment are readily adsorbed by leaf tissues, further compromising the inherent quality of fresh leaves. Airtight storage and transport containers promote heat accumulation at the core of leaf piles, accelerating leaf reddening and browning reactions. In addition, mechanical vibration and impact during transportation induce physical injury to leaf tissues, which activates endogenous enzyme activity at damaged sites, triggers premature oxidation reactions, and exacerbates fresh-leaf deterioration.

Controlled storage and transportation trials demonstrate that an ambient temperature of 30 °C markedly exacerbates water loss and browning in fresh leaves, whereas low-temperature storage and transport at 8 °C effectively reduce leaf metabolic intensity, mitigate multiple forms of physical damage, and preserve the intact morphology and basal quality of fresh tea leaves [2]. Physical damage to fresh leaves further triggers subsequent biochemical deterioration, representing a predisposing factor for quality degradation in the final processed tea products. Accordingly, the stringent regulation of fresh-leaf storage and transportation conditions constitutes the primary upstream step in full-chain tea preservation.

### 2.2. Physical Deterioration Profiles of Processed Tea During Storage

Processed tea features a loose tissue structure, well-developed porous architecture, and high specific surface area, conferring strong adsorptive and hygroscopic properties. These inherent characteristics represent the core drivers of apparent physical deterioration in tea during storage. Under conventional storage environments, tea readily adsorbs water vapor, exogenous odors, and particulate matter from ambient air, leading to the elevated moisture content of dry tea, a softened texture, and diminished surface cleanliness. These changes create favorable prerequisites for microbial proliferation and subsequent biochemical reactions. Volatile aroma compounds in tea, including terpenes, alcohols, and aldehydes, exhibit relatively low boiling points. Over prolonged storage, these compounds undergo continuous volatilization and dissipation, leading to gradual fading and dulling of the rich, fresh aroma profile of tea. Concurrently, sustained exposure to light and oxygen induces color degradation in tea. The vibrant yellow–green hue of green tea, the delicate aroma of scented tea, and the overall luster of dry tea all diminish under such conditions, resulting in substantial reduction in the commercial value of tea products. Relative to biochemical deterioration, physical deterioration is visually perceptible and persistent and serves as the primary basis for consumer assessment of tea freshness.

### 2.3. Biochemical Deterioration Mechanism of Processed Tea During Storage

Biochemical reactions constitute the core intrinsic cause of irreversible quality deterioration in tea during storage. Metabolic imbalance and structural disruption of diverse bioactive constituents in tea underpin the fundamental mechanisms of tea aging and quality degradation. The deterioration process is centered on tea polyphenol transformations, accompanied by sequential metabolic alterations in tea pigments, amino acids, lipids, and aroma compounds. All constituent classes are interrelated and collectively drive tea quality deterioration.

#### 2.3.1. Oxidation of Tea Polyphenols

Tea polyphenols represent the most abundant characteristic flavor and functional substances in tea with the strongest bioactivity. Among these compounds, oxidative degradation of catechins constitutes the core biochemical reaction underlying tea aging and quality deterioration. During green tea storage, changes in tea polyphenol content are highly synchronized with deterioration trends in sensory attributes including infusion color, taste, and aroma. A 5% reduction in tea polyphenol content induces a marked decline in tea quality. When the reduction reaches 25%, the inherent flavor profile of tea is largely lost, along with its drinking and commercial value [13].

Under the combined action of endogenous enzymes and oxygen, catechins undergo continuous oxidation and polymerization to form dark-colored secondary products, including theabrownins. These compounds not only deepen the color of tea infusion and leaf base but also disrupt the mellow taste and astringency balance of the infusion, resulting in taste profile imbalance [13]. At the individual compound level, catechins positively associated with fresh flavor attributes, including EGC, EGCG, and ECG, decline continuously during green tea storage, whereas deterioration-related constituents such as GC and EC accumulate progressively. The resulting imbalance in catechin proportions further exacerbates the flavor quality degradation in tea. Multi-omics investigations of storage and transportation processes corroborate this pattern. An ambient temperature of 30 °C accelerates degradation of EGC, EC, EGCG, and ECG, and promotes aberrant enrichment of GC and C. Low-temperature conditions at 8 °C effectively preserve galloylated catechins, whereas flavonoids exhibit concurrent degradation and differential enrichment. Key genes including SCPL, ANR, and LAR are implicated in this metabolic regulatory process [2]. The metabolic patterns of tea polyphenols differ substantially across tea categories. The flavor profile of dark tea during storage is primarily determined by flavonol glycosides and polymerized catechin derivatives, the contents of which are modulated by storage relative humidity [8]. During white tea storage, catechins and the majority of amino acids decrease progressively, while 8-C *N*-ethyl-2-pyrrolidinone-substituted flavan-3-ols (EPSFs) and pyroglutamic acid accumulate continuously [9]. Among these compounds, EPSFs serve as characteristic markers for determining the storage age of white tea [14]. In addition, storage temperature and humidity significantly influence the physicochemical stability of amorphous EGCG. Water vapor induces recrystallization of the compound, low humidity readily causes oxidative discoloration, and moderate humidity exacerbates its epimerization reaction [15].

#### 2.3.2. Transformation of Tea Pigment Components

Tea pigments comprise water-soluble fractions including theaflavins, thearubigins, and theabrownins, as well as fat-soluble chlorophyll, all of which represent key determinants of tea color attributes. During storage, theaflavins and thearubigins in black tea and white tea are susceptible to oxidative polymerization and gradual conversion to theabrownins, resulting in darker infusion color and diminished brightness. Black tea exhibits a short-term post-ripening effect during storage. The contents of theaflavins and thearubigins show an initial increase followed by a subsequent decrease, whereas theabrownins accumulate continuously throughout the period. Higher ambient temperatures accelerate pigment imbalance and induce more pronounced tea quality deterioration [10].

Chlorophyll constitutes the core pigment responsible for the color of green tea dry leaves and infusions, and exhibits high sensitivity to temperature, moisture, and light. During storage, chlorophyll readily undergoes demagnesiumation and decomposition reactions, converting from a vibrant emerald green to dark brown pheophytins. Previous investigations have confirmed that the chlorophyll conversion rate directly determines the color quality of green tea [11]. When the demagnesiumation conversion rate of chlorophyll remains below 10%, green tea largely retains its original color. When the conversion rate exceeds 70%, severe browning occurs, and the appearance quality of premium green tea is completely compromised. Following one year of storage under ambient-temperature conditions, total chlorophyll content in tea may decrease by 12.79%, with chlorophyll a exhibiting a significantly higher degradation rate than chlorophyll b [11].

In postharvest fresh leaves, by contrast with the storage-driven chlorophyll degradation in processed green tea described above, the storage and transportation temperatures of fresh leaves also modulate the chlorophyll metabolic pathway. Ambient temperature upregulates genes associated with chlorophyll degradation, whereas low-temperature conditions at 15 °C suppress the expression of *CLH* and *NYE1*. When combined with low-concentration CO_2_ modified atmosphere packaging, the green color retention effect is further enhanced. Low-temperature conditions also elevate the chlorophyll fluorescence parameter Fv/Fm and preserve the appearance quality of fresh leaves [2,16]. Chlorophyll content serves as a reliable indicator for assessing green tea freshness and storage duration, and its irreversible degradation represents one of the core factors limiting the shelf life of premium green tea [5,17].

#### 2.3.3. Degradation and Transformation of Amino Acids

Amino acids represent core constituents responsible for the fresh and mellow taste of tea infusions, and their absolute contents and relative proportions directly influence the flavor performance of tea products. In freshly processed tea, L-amino acids constitute the predominant enantiomeric form. During prolonged storage, theanine undergoes chiral isomerization to form D-amino acids. Extended storage duration correlates with increased D-amino acid content and elevated D/L enantiomeric ratios in tea [18,19].

Tea amino acids exhibit limited chemical stability, and two primary deterioration pathways have been identified during storage. First, amino acids may react with polyphenols to form dark brown insoluble polymers, which darken tea infusion and reduce its clarity. Second, amino acids participate in Maillard reactions with soluble sugars to generate browning products, leading to dull color and diluted taste. This process represents a major contributor to the loss of fresh, mellow flavor in stored tea. For high-quality green tea stored without preservation interventions, amino acid content declines markedly within the first six months, followed by disruption of constituent proportions. These changes completely alter the inherent flavor profile of the product [5]. For postharvest fresh leaves rather than stored processed tea, storage and transportation investigations reveal that non-refrigerated, ambient-temperature transportation induces aberrant enrichment of branched-chain amino acids, including valine and isoleucine. This represents a typical signature of amino acid metabolic disorder in fresh leaves under high-temperature stress. While low-temperature storage and transportation can mitigate this phenomenon, they cannot completely prevent theanine degradation [2]. Collectively, amino acids not only dominate the taste profile of tea infusions but also exert important regulatory effects on tea color stability. Their degradation degree can serve as a key indicator for evaluating the storage quality of tea products.

#### 2.3.4. Oxidative Rancidity of Lipids

The general chemistry of unsaturated fatty acids oxidation, characterized in model oil systems [20], shows that these compounds serve as critical precursors for delicate aroma profiles yet exhibit limited chemical stability and are susceptible to hydrolysis, oxidation, and rancidification during storage. Tea lipids consist predominantly of unsaturated fatty acids including linolenic acid and linoleic acid, along with carotenoids. Both classes undergo autoxidation during storage to generate low-molecular-weight off-flavor compounds including aldehydes, ketones, and alcohols. In edible oil systems, such oxidation products have been linked to stale and stuffy off-odors in aged tea [21], and a comparable contribution is plausible in aged tea, though the tea-specific validation of these pathways remains limited and direct extrapolation from oil-system kinetics should be interpreted with caution given the distinct matrix composition, moisture level, and coexisting polyphenol antioxidants in tea.

In green tea, the GC-MS analysis of Huangshan Maofeng green tea stored at 25 °C for up to 150 days detected nine fatty acids including palmitic, stearic, oleic, linoleic, and linolenic acids, most of which declined sharply after 60 days of storage, with the total saturated and unsaturated fatty acid contents decreasing progressively and correlating closely with sensory freshness scores [22]. Among these, linolenic acid content exhibits a significant positive correlation with tea freshness. During Pu-erh tea storage, terpene aroma compounds undergo structural rearrangement, generating numerous isomers and leading to fading of the original aroma profile. Accordingly, the dysregulation of lipid metabolism represents a shared mechanism underlying aroma deterioration across diverse tea categories. Low-temperature conditions preserve tea lipid constituents and reduce oxidative rancidification as well as the formation of off-flavor compounds in postharvest fresh leaves [2], a finding consistent with but not identical to the lipid stabilization observed in stored processed green tea.

#### 2.3.5. Evolution of Aroma Components

Tea aroma compounds are primarily synthesized via terpenoid, phenylpropanoid, lipoxygenase, and other metabolic pathways. Postharvest temperature modulates gene expression across these related pathways, thereby altering the composition of aroma constituents [23]. The evolutionary patterns of aroma compounds following storage differ markedly across distinct tea categories [5]. An ambient temperature of 25 °C exacerbates metabolic breakdown of aroma precursors, whereas low-temperature conditions at 15 °C stabilize the expression of associated genes and delay the loss of high-quality aroma compounds. This regulatory effect constitutes the intrinsic mechanism by which low temperature preserves the original fresh aroma of tea.

Flavor constituents in green tea undergo substantial changes during storage. Characteristic floral compounds including linalool and nerol are readily lost, while stale off-flavor compounds such as propionaldehyde and 1-penten-3-ol form progressively. Concurrently, sweet and floral compounds including benzyl alcohol and β-ionone increase in content, leading to the gradual weakening of the fresh, clean aroma profile [24]. The investigations of Longjing tea demonstrate that high temperature and aerobic conditions accelerate the oxidation of lipids and carotenoids, generating stale off-flavor compounds with woody and greasy notes, including ketones and alcohols. The application of oxygen scavengers effectively inhibits this oxidation process and reduces the formation of off-flavors [25]. Characteristic aldehyde aroma compounds in oolong tea, including hexanal and 2-methylbutanal, are susceptible to oxidative loss under ambient-temperature conditions. Reduced glutathione stabilizes these volatile compounds via molecular binding interactions and mitigates aroma deterioration [12]. For deeply processed tea products such as instant tea, prolonged storage readily induces loss of volatile aroma compounds. Microencapsulation technology forms a protective barrier via wall materials, effectively reducing aroma volatilization and oxidative decomposition to achieve long-term aroma retention [26].

Relative to constituents such as tea polyphenols and chlorophyll, aroma compounds in green tea exhibit higher sensitivity to storage environmental conditions. Among these compounds, contents of alcohols including linalool are positively correlated with tea freshness, whereas abundant generation of aldehydes and ketones such as (*E*,*E*)-2,4-heptadienal, α-terpineol, (*E*,*E*)-2,4-nonadienal, and (*E*,*E*)-2,4-decadienal represent a typical signature of staleness in green tea [27,28]. Additionally, furosine and 5-hydroxymethylfurfural serve as characteristic markers of Maillard reactions in tea. Their contents increase under both ambient and high-temperature storage conditions and can be applied in combination for quality monitoring across the entire processing and storage cycle of tea [29].

### 2.4. Correlation Between Deterioration Products and Sensory Quality of Tea

Storage-induced deterioration of green tea represents a comprehensive outcome of oxidation, degradation, and transformation of multiple endogenous constituents. Significant quantitative correlations exist between biochemical indices and sensory attributes including color, taste, and aroma, with distinct chemical components contributing differentially to specific sensory qualities.

Using Biluochun tea as the research material, scholars systematically investigated dynamic changes in eight indices during storage, including moisture content, caffeine, amino acids, total soluble sugar, chlorophyll, tea polyphenols, water-soluble extracts, and ascorbic acid. Correlation and regression analyses were subsequently conducted in combination with sensory evaluation scores. The results demonstrate that the correlation coefficient between the oxidative degradation degree of ascorbic acid and the sensory quality deterioration of Biluochun tea reaches 0.992, identifying ascorbic acid as a core indicator for characterizing green tea storage quality. A prediction model constructed based on this index, expressed as y = 32.447 + 305.390x where y represents quality score and x represents ascorbic acid content, enables the accurate prediction of green tea quality changes during storage [30].

Overall, tea polyphenols, amino acids, and soluble sugars collectively determine the freshness and sweet mellow taste of tea infusions. The degradation of these constituents directly results in a weak taste intensity and unbalanced bitter-astringent ratios. Chlorophyll constitutes the core pigment, maintaining the emerald green color of green tea dry leaves, infusions, and leaf bases, and its degradation represents the primary cause of tea color deterioration. Intermediate products generated by ascorbic acid oxidation can induce carbonyl-amine browning, further exacerbating a dull coloration and freshness loss in tea [30]. The combined effects of multiple reactions ultimately result in the comprehensive sensory quality degradation of green tea across color, aroma, and taste attributes.

### 2.5. Alterations in Harmful Substances During Storage

Tea storage not only induces deterioration of flavor and nutritional constituents but also drives dynamic changes in certain harmful substances, which are directly associated with food safety. Current research on harmful substances in stored tea primarily focuses on typical processing and storage pollutants, including polycyclic aromatic hydrocarbons (PAHs) and acrylamide. PAH pollutants in dark tea undergo natural degradation during storage, with storage temperature significantly influencing degradation efficiency [31]. Comparison of three storage conditions, namely ambient temperature, 4 °C, and −20 °C, reveals that the 4 °C low-temperature environment achieves the highest efficiency in reducing PAH residues. Ambient temperature favors the degradation of low-ring PAHs, whereas low-temperature conditions exhibit higher efficacy for degrading high-ring PAHs. All treatment groups display faster degradation rates during the initial storage phase [31]. With extended storage duration, quality constituents and antioxidant activities of Wuyi rock tea, including tea polyphenols, catechins, and caffeine, decline progressively, while total soluble sugar content increases. By contrast, acrylamide content decreases continuously with storage, with all sample concentrations remaining below EU safety limits, indicating good drinking safety [32]. Notably, current studies on harmful-substance evolution are limited to only a few tea categories, and quantitative relationships between storage conditions and pollutant levels have not been fully established.

Overall, postharvest quality deterioration of tea shares a universal core reaction backbone but presents prominent category-specific divergence patterns. Oxidative degradation of endogenous components serves as the fundamental mechanism across all tea categories, driven by three core environmental factors of moisture, temperature and oxygen, with polyphenol oxidation, pigment transformation and lipid rancidity as the shared reaction pathways. Shaped by differences in fermentation degree, residual enzyme activity and matrix composition, the dominant deterioration modes fall into three distinct paradigms. Unfermented and lightly fermented teas are dominated by oxidation of heat-labile components with the highest environmental sensitivity, and the long-standing debate over optimal storage temperature essentially reflects a trade-off between preservation efficacy and energy cost rather than contradictory mechanistic conclusions. Semi-fermented and fully fermented teas exhibit a dual-phase pattern of early post-ripening quality improvement followed by late quality decline, with their optimal storage window closely tied to initial fermentation level and roasting intensity. For post-fermented teas, microbially mediated substance transformation runs through the entire storage process, where moderate oxygen and humidity act as necessary conditions for flavor maturation rather than purely harmful factors; the lack of quantitative standards for this moderate interval remains the core bottleneck restricting the standardized aging of post-fermented tea. Notably, postharvest fresh-leaf deterioration follows the regulatory logic of living tissue physiological metabolism centered on respiratory water loss and stress browning, which is fundamentally distinct from the oxidative polymerization pathway in processed tea. Confusion between these two deterioration systems is a key reason for the poor industrial adaptability of many laboratory-derived process parameters.

## 3. Key Environmental Factors Governing Postharvest Quality Deterioration of Tea

**Figure 1:**
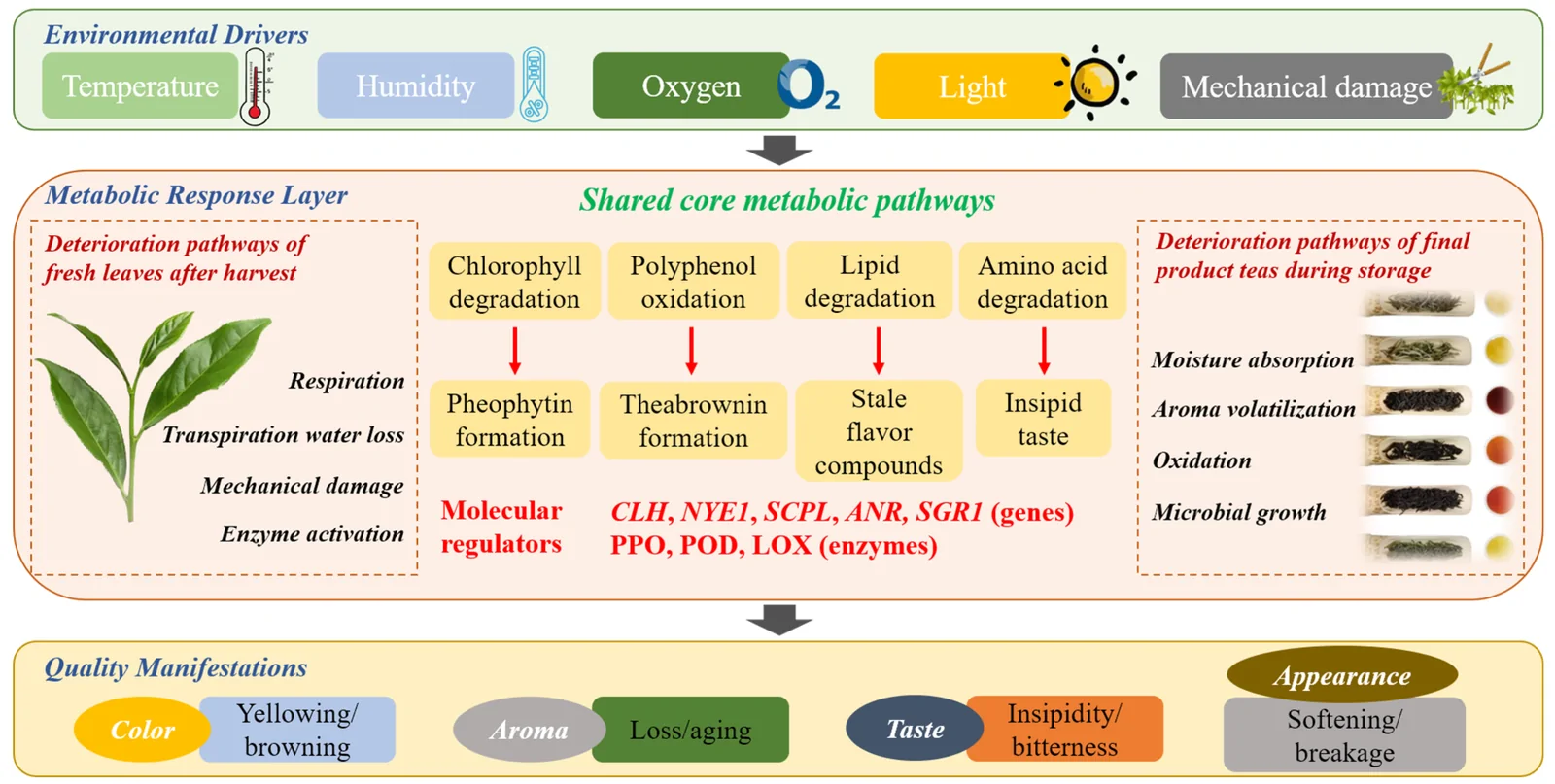
Multi-dimensional regulatory mechanism of postharvest quality deterioration in the whole tea chain. Schematic representation of coupled deterioration processes in fresh leaves and finished tea, illustrating how environmental factors regulate endogenous metabolic pathways to drive overall quality degradation. PPO, polyphenol oxidase; POD, peroxidase; LOX, lipoxygenase; *CLH, chlorophyllase gene; NYE1, non-yellow coloring 1; SCPL, serine carboxypeptidase-like gene; ANR, anthocyanidin reductase gene; SGR1, non-yellow coloring 1/stay-green 1*.

The postharvest quality deterioration of tea arises from the synergistic interplay of physical, chemical, and biological processes. Ambient environmental factors serve as the core driving forces that trigger and exacerbate the metabolic dysregulation of endogenous constituents and subsequent quality degradation in tea products. Moisture, oxygen, temperature, and light represent four pivotal regulatory factors governing tea storage quality. These factors modulate the profile of endogenous constituents in tea via the regulation of enzymatic reactions, accelerating oxidative metabolism, and promoting compound degradation, which collectively culminate in sensory quality deterioration. The multi-dimensional regulatory network underlying whole-chain tea quality deterioration is visually summarized in Figure 1, which illustrates the coupling effects of endogenous metabolic pathways and exogenous environmental factors across the entire postharvest process. Meanwhile, packaging materials and storage vessels, as critical determinants of the storage microenvironment, exert indirect yet substantial effects on tea quality dynamics and constitute an indispensable supporting component of tea preservation systems.

### 3.1. Moisture

Moisture represents the primary contributor to tea quality deterioration and functions as the fundamental solvent for all biochemical reactions, directly governing the rate of tea aging and overall storage stability. The inherent porous architecture of tea tissues confers pronounced hygroscopic properties. Exposure to high-humidity environments induces a rapid elevation in the moisture content of dried tea, which activates endogenous enzymes including polyphenol oxidase and peroxidase, accelerates the oxidative polymerization of tea polyphenols and hydrolytic rancidification of lipids, and consequently induces quality defects including a darkened infusion, altered astringency profiles, and pronounced stuffy off-odors. Concurrently, elevated moisture conditions promote the proliferation of deleterious microorganisms including molds and bacteria, which may result in mold spoilage and complete loss of commercial value.

Empirical industrial practices and scientific investigations have consistently demonstrated that the safe moisture content for dried tea storage should be maintained within the range of 4% to 6%. Within this interval, endogenous enzyme activity and non-enzymatic oxidation reactions in tea are effectively suppressed, yielding optimal quality stability. Moisture contents above 6.0% accelerate quality degradation, and levels exceeding 7.0% substantially increase the risk of microbial proliferation and mold spoilage. The regulation of water activity represents an effective strategy to delay quality deterioration in green tea [4]. Conversely, moisture contents below 3% predispose tea to the development of a high-fire off-flavor, which compromises its inherent flavor profile [33]. The tolerance thresholds for storage moisture content and ambient humidity vary considerably across tea categories [5]. Premium green tea exhibits the highest sensitivity to moisture. At moisture contents exceeding 7%, conventional preservation strategies are largely ineffective at maintaining freshness, and levels reaching 10% pose an extremely high risk of spoilage. In black tea, elevated moisture contents correlate with more pronounced losses of theaflavins and thearubigins, as well as progressive declines in infusion freshness and stability. Comparative trials have verified that high-humidity environments exacerbate both polyphenol degradation and attenuation of antioxidant activity in black and green tea. Green tea exhibits greater susceptibility to humidity-induced changes, whereas black tea demonstrates higher hygroscopicity and relatively greater stability of its polyphenol system under high-humidity conditions [7]. Moisture also exerts pronounced effects during the storage and transportation phase. The water loss rate of fresh leaves during long-distance ambient-temperature transportation is substantially higher than that under low-temperature conditions. Low-temperature treatment reduces transpiration-mediated water loss in leaves and preserves favorable moisture status in fresh tea leaves [2].

For dark tea, ambient relative humidity represents a key regulator of metabolite profiles and microbial community structure. A relative humidity of approximately 57% sustains high levels of catechin polymers and flavonoids in raw dark tea, corresponding to optimal comprehensive sensory quality. Genera including *Aspergillus*, *Gluconobacter*, *Kluyveromyces*, and *Pantoea* constitute the core functional microbiota during dark tea storage. Humidity governs substance transformation and flavor formation primarily via modulating microbial community structure [8]. In Pu-erh tea, higher moisture contents accelerate polyphenol transformation. However, the combination of excessive humidity and low temperature induces massive proliferation of molds and yeasts, which markedly increases the risk of storage-related quality deterioration [34]. Collectively, moisture control constitutes the primary defensive barrier for long-term tea preservation.

### 3.2. Oxygen

Oxygen represents an essential prerequisite for the oxidative deterioration of endogenous tea constituents. Its influence persists throughout the entire storage cycle and exerts a decisive effect on tea quality stability. Oxygen directly participates in the oxidative metabolism of tea polyphenols, unsaturated fatty acids, and volatile aroma compounds, leading to the progressive depletion of flavor and functional constituents and inducing tea browning, stale flavor development, and aroma loss. Furthermore, oxygen-mediated oxidation processes are persistent and irreversible. Tea aging reactions proceed continuously as long as residual oxygen remains present.

Investigations conducted on Longjing tea demonstrate that oxygen accelerates lipid and carotenoid oxidation under high-temperature conditions, leading to the formation of diverse stale off-flavor compounds. The removal of oxygen from packaging via oxygen scavengers significantly mitigates high-temperature-induced flavor degradation and preserves the inherent floral and chestnut aroma profiles of tea [24,25]. Notably, oxygen requirements differ substantially across tea categories. Most tea types, including green tea and black tea, require oxygen exclusion to delay quality degradation, whereas flavor development in post-fermented teas such as dark tea depends on moderate oxygen availability to facilitate substance transformation [5]. To maximally inhibit both enzymatic and non-enzymatic oxidation reactions, conventional hermetic tea packaging requires strict control of oxygen content below 0.1% [4]. Roasted green tea stored in conventional packaging at ambient temperature exhibits accelerated depletion of endogenous constituents due to continuous oxygen exposure. Vacuum packaging, which achieves oxygen isolation, effectively delays its quality deterioration [35]. Building on the mechanistic understanding of oxygen-induced deterioration, modified atmosphere preservation strategies including low-oxygen conditioning, nitrogen flushing, and deoxygenation have emerged as mainstream preservation approaches for high-end tea products.

### 3.3. Temperature

Temperature governs the kinetics of biochemical reactions. Elevated temperatures accelerate the degradation of heat-labile constituents and the transformation of pigment compounds. For finished green tea products, each 10 °C elevation in ambient temperature increases the quality degradation rate by a factor of 3 to 4. A temperature of 4 °C represents the preferred condition for conventional commercial storage, while ultra-low-temperature storage at −10 to −15 °C substantially extends shelf life, albeit with markedly elevated energy consumption and operational costs [4]. Optimal storage temperatures vary across tea categories, and lower temperatures do not necessarily translate to superior preservation efficacy [5]. Refrigeration at 0 to 10 °C effectively slows the degradation of theaflavins and thearubigins as well as the formation of theabrownins. Nevertheless, if the initial moisture content of black tea is elevated, pigment degradation still proceeds at an accelerated rate even under low-temperature conditions [10]. Longjing tea exhibits minimal quality variation under low-temperature conditions of −20 °C, −5 °C, and 4 °C, whereas ambient temperature at 25 °C accelerates the loss of chlorophyll, ascorbic acid, and other constituents, leading to sensory quality degradation [36]. In long-term storage scenarios, frozen storage of green tea at −18 °C achieves superior preservation efficacy compared with refrigeration at 0 to 5 °C. Light-flavor oolong tea demonstrates higher temperature sensitivity, for which frozen storage is preferentially recommended [37]. The storage characteristics of Wuyi rock tea differ substantially from those of green and black tea. Long-term storage at 5 °C retains roasted and astringent flavor notes, whereas storage at ambient temperatures ranging from 3 °C to 30 °C gradually diminishes roasted off-notes, enriches aroma complexity, and yields a mellow, sweet taste profile. This pattern aligns with the characteristic aging behavior of rock tea [38]. Elevated temperatures accelerate endogenous substance transformation in Pu-erh tea and shorten its aging cycle. However, the combination of 30 °C and high-humidity conditions predisposes the product to mold growth, adverse storage off-flavors, and quality defects. This necessitates complementary moisture control management to mitigate associated risks [34]. Albino green tea is prone to the formation of stale volatile compounds under high-temperature conditions. Experimental calculations indicate its theoretical shelf life at 20 °C is 320 days [39]. Matcha exhibits pronounced temperature sensitivity. Elevated temperatures accelerate the degradation of chlorophyll, catechins, polyphenols, flavonoids, and other constituents, and 4 °C represents a suitable temperature for long-term matcha storage [40]. Temperature also influences the flavor performance of tea beverages. Storage at 37 °C alters the constituent profile and aroma type of Dahongpao oolong tea beverages, whereas low-temperature storage at 4 °C better preserves their inherent flavor characteristics. Carotenoid metabolites have been identified as the key compounds driving these flavor differences [41].

For freshly harvested tea leaves, ambient temperature exacerbates physiological metabolism and accelerates flavor constituent loss, whereas low-temperature treatment at 15 °C effectively reduces metabolic intensity and mitigates aroma compound loss, representing a suitable strategy for short-term preservation of fresh tea leaves [23]. From molecular and storage–transport mechanistic perspectives, simulated transportation trials conducted on the fresh leaves of Yinghong No. 9 demonstrate that high-temperature treatment at 30 ± 3 °C reduces leaf Fv/Fm values, elevates the browning index, and induces the aberrant metabolism of branched-chain amino acids and galloylated catechins. Low-temperature conditions at 8 °C modulate the expression of TCS, a key gene in the caffeine biosynthetic pathway, and maintain metabolic homeostasis [2]. Fresh-leaf withering trials verify that a low-temperature treatment at 15 °C suppresses the expression of key chlorophyll degradation genes including CLH and NYE1. When combined with high-concentration CO_2_ temperature control, this treatment further enhances green color retention efficacy [16].

Tea products removed from cold storage are susceptible to condensation and moisture absorption due to temperature differentials. A 35 °C hot-air temperature compensation process effectively mitigates this issue [11,42]. It is noteworthy that standalone low-temperature storage is associated with several limitations, including high energy consumption, susceptibility to exogenous odor adsorption in storage facilities, and condensation induced by temperature differentials during product removal. In practical applications, it must be implemented in conjunction with hermetic odor-resistant packaging and gradual temperature acclimation procedures. From an industrial application perspective, low-temperature storage extends the shelf life of premium green and oolong teas by 6 to 12 months, representing the most mature technology with established cold-chain infrastructure, though its high energy cost limits adoption for mid- and low-value teas. Storage temperature exerts significant effects on endogenous constituents and antioxidant activity of green tea. Ambient temperature at 25 °C accelerates the degradation of key components including catechins and amino acids, thereby exacerbating quality degradation [43]. Furthermore, combined high temperature and high humidity accelerate the racemization of theanine in green tea, whereas low-temperature, low-humidity environments favor the retention of natural L-theanine [18].

### 3.4. Light

Light represents a key inducer of photooxidative deterioration in tea, exerting potent destructive effects on pigments, lipids, and aroma compounds. Light-induced quality damage is irreversible [5]. Ultraviolet and visible wavelengths in natural light directly degrade chlorophyll and disrupt polyphenol structure, while simultaneously catalyzing the oxidation of unsaturated fatty acids. These effects not only induce the yellowing of green tea and loss of luster in dried tea leaves but also generate off-flavors such as sun-dried taste notes. Accordingly, roasted green tea requires full light exclusion throughout the entire storage process [35]. Ultraviolet radiation in natural light exerts far greater oxidative damage to pigments and lipids than visible light, and constitutes the primary wavelength band driving photooxidative deterioration.

Indoor simulation trials demonstrate that continuous irradiation of green tea with 1700 lx fluorescent light for 30 days at 25 °C completely eliminates the green hue of tea and induces severe quality degradation. Light also accelerates lipid photooxidation, leading to the formation of aldehyde and ketone off-flavors and complete disruption of the inherent tea flavor profile [4]. Accordingly, full light exclusion represents not only a fundamental requirement for tea storage and preservation but also a core design criterion for tea packaging.

### 3.5. Packaging and Storage Vessels

Packaging materials and storage vessels serve as critical barriers against external moisture, oxygen, light, and exogenous odors, and play a central role in stabilizing the storage microenvironment. Their material composition and structural design directly determine preservation efficacy and flavor evolution trends in tea.

#### 3.5.1. Packaging Materials

High-quality tea packaging must simultaneously satisfy core requirements for moisture resistance, oxygen barrier performance, light exclusion, and hermetic sealing. The lower oxygen and water vapor permeability of packaging materials correspond to superior barrier performance [5]. The combination of high-barrier hermetic packaging and low-temperature environments maximally preserves the aroma and taste profile of green tea and delays quality degradation throughout the shelf-life period. Small-format nitrogen-flushed packaging reduces internal oxygen concentration and effectively inhibits oxidative deterioration of tea, which is the most widely adopted packaging format for commercial tea products at present [44]. Conversely, non-hermetic packaging exposes tea to continuous moisture, oxygen, and light exposure, accelerating the degradation of active constituents including polyphenols and flavonoids and diminishing its antioxidant and antibacterial activities. Hermetic packaging effectively mitigates these adverse effects [45].

The application suitability of different plastic and composite packaging materials varies considerably. Packaging materials including polyethylene, polyethylene terephthalate (PET), and aluminum–plastic composites exhibit low adsorption affinity for green tea aroma compounds and low propensity for harmful-substance migration, demonstrating high application suitability. Polypropylene packaging may release off-flavor compounds such as 2,4-*di*-*tert*-butylphenol, which compromises tea flavor quality [46]. Under light exposure conditions, glass and paper packaging accelerate chlorophyll degradation in green tea, whereas metal cans and original factory packaging effectively delay pigment loss [17]. Storage trials conducted on Lapsang Souchong black tea verify that aluminum foil-kraft paper composite bags achieve the best comprehensive barrier performance, effectively slowing moisture content elevation and degradation of tea polyphenols and free amino acids. Pure aluminum foil bags and kraft paper bags exhibit intermediate performance, while corn fiber bags demonstrate the poorest preservation efficacy. Nevertheless, single packaging materials have limited efficacy in retarding overall black tea deterioration, and require combination with other preservation technologies under high-temperature conditions [47]. In industrial practice, multi-material composite packaging is widely adopted, with customized solutions developed according to specific tea characteristics to maximize tea quality protection.

#### 3.5.2. Storage Vessels

For post-fermented teas such as ripe Pu-erh tea, air-permeable storage vessels including pottery jars, metal tins, cartons, and wooden boxes significantly enhance infusion sweetness, intensify aged aroma, and reduce bitterness compared with original factory hermetic packaging [48]. 1,2,4-Trimethoxybenzene has been identified as a characteristic volatile marker for differentiating ripe Pu-erh stored in different vessels. Distinct container types impart differentiated flavor profiles to ripe Pu-erh, including woody and jujube-like aroma notes. Packaging and storage vessels represent integral components of the storage microenvironment. Their barrier performance and material properties indirectly modulate ambient conditions including moisture, oxygen, and light, thereby either accelerating or delaying tea quality deterioration.

Table 2 outlines the reported recommended storage environmental parameters and corresponding packaging requirements for representative tea categories, providing a practical reference for standardized industrial operation. Collectively, the four major environmental factors of moisture, oxygen, temperature, and light act via coupled and synergistic mechanisms to jointly govern the tea deterioration process [3,13]. Distinct tea categories require matched environmental control strategies tailored to their inherent characteristics, which also provides an important foundation for subsequent preservation technology research and development. Optimal storage conditions for black tea involve temperatures of −10 to −5 °C, a moisture content of approximately 3%, and vacuum deoxygenation combined with aluminum foil bag packaging. This combination maximally preserves tea polyphenol content and maintains tea sensory quality [49]. For raw Pu-erh tea, endogenous constituents and tea infusion pH exhibit greater stability under constant temperatures ranging from 5 °C to 20 °C. A high temperature of 35 °C exacerbates component fluctuations but exerts no significant effect on tea infusion color [50]. Moisture and chlorophyll contents in matcha are highly correlated with sensory quality and can serve as core indicators for shelf-life prediction. Low-temperature storage represents an effective strategy to delay its quality degradation [51]. For raw Pu-erh tea, temperature and humidity significantly alter its metabolic profile and aroma characteristics. Dry-cold storage favors the retention of floral and fruity sweet aroma notes, whereas hot-humid environments promote the development of aged aroma and mellow taste profiles. These two storage environment types correspond to distinct characteristic aroma markers, which can provide a basis for the optimization of Pu-erh storage technologies [52,53]. Against the current developmental backdrop of the tea industry, single-factor environmental control or standalone packaging approaches can no longer satisfy the storage requirements of high-quality tea. The research and development of green, precise, and composite preservation technologies, along with the establishment of a whole-process standardized storage management and control system, represent key directions for reducing circulation losses, enhancing the commercial value of tea products, and promoting high-quality development of the tea industry.

## 4. Mainstream Postharvest Preservation Technologies for Tea Products

Tea preservation constitutes a full-chain systematic endeavor encompassing fresh-leaf harvesting, cold-chain transportation, spreading and withering, pre-processing treatment, finished product storage, and terminal distribution. To date, the majority of scholarly investigations have centered on storage and preservation technologies for finished tea products, whereas research on source-end preservation of postharvest fresh leaves remains comparatively limited. Nevertheless, given that fresh-leaf quality underpins the final quality of processed tea, effective preservation of postharvest fresh leaves mitigates raw material deterioration at the source and stabilizes downstream product quality, conferring substantial research and applied value. Pre-cooling, short-term preservation, and standardized storage and transportation of postharvest fresh leaves represent the primary upstream components of full-chain tea quality management.

Drying pretreatment serves as a foundational prerequisite for long-term tea preservation. Through unit operations including fixation and re-drying, the moisture content of dried tea is maintained within the safe storage range of 4% to 6%. This practice substantially inactivates endogenous enzyme activity, suppresses microbial proliferation, fundamentally reduces the rate of biochemical deterioration, and establishes stable operational conditions for diverse preservation strategies, including physical, modified atmosphere, and biological preservation technologies. Currently, industrial tea preservation has developed into a diversified technical framework, encompassing low-temperature refrigeration, drying and humidity regulation, preservative application, modified atmosphere deoxygenation, irradiation, packaging-based preservation, and biological preservation. As all standalone preservation technologies exhibit inherent limitations, multi-technology synergistic strategies have emerged as the mainstream trend in industrial research and technological upgrading.

### 4.1. Preservation Technologies for Storage and Transportation of Fresh Tea Leaves

Postharvest fresh tea leaves maintain active physiological metabolism and are highly susceptible to environmental stressors, including temperature, humidity, mechanical vibration, and oxygen exposure. These factors induce adverse outcomes such as water loss and wilting, temperature elevation and reddening within leaf piles, and mold spoilage. Such quality degradation not only compromises the final quality of processed tea but also constrains the large-scale storage, transportation, and standardized processing of tea raw materials [54]. Temperature serves as the core control parameter for fresh-leaf storage and transportation. A constant temperature ranging from 15 °C to 18 °C effectively preserves fresh-leaf quality within a 24 h timeframe. For storage durations exceeding 24 h, humidity regulation is necessary, and a relative humidity of 85% to 90% further decelerates the deterioration process of fresh leaves [55]. Comparative trials demonstrate that low-temperature transportation at 10 °C reduces the mass loss rate of fresh leaves by 2.3% to 2.5% relative to ambient-temperature transportation within a 6 h storage and transportation window, yielding significant temperature-controlled preservation efficacy [54].

Furthermore, mechanical vibration exacerbates fresh-leaf injury. Mechanical impact during transportation accelerates polyphenol oxidation and elevates the risk of leaf reddening and mold spoilage, and these adverse effects are further amplified under high-temperature conditions [55,56]. In addition, the structural design and ventilation performance of storage containers directly influence the heat dissipation efficiency of leaf piles. Conventional airtight transport baskets readily induce overheating at the core of leaf piles, whereas ventilated transport baskets reduce the core temperature of leaf piles by 4 °C to 5 °C after 4 h of storage and transportation relative to traditional baskets [54]. Oxygen concentration exerts minimal effects on short-term storage and transportation of fresh leaves. Accordingly, targeted oxygen regulation is unnecessary for conventional large-scale operations, which contributes to reduced preservation costs [55].

From the perspective of chlorophyll metabolism, low-temperature storage and transportation at 15 °C downregulates the expression of genes associated with chlorophyll degradation and reduces the formation of browning products. The integrated strategy combining low temperature, light exclusion, and moderate CO_2_ modified atmosphere represents the optimal protocol for fresh-leaf storage and transportation. A low-temperature environment with yellow light illumination also enhances the accumulation of floral aroma compounds in tea while exerting color-protective effects [16]. Investigations conducted by Mei et al. [57] on the fresh leaves of Yinghong No. 9 further corroborated that low-temperature and high-humidity conditions effectively preserve the moisture content and photosynthetic activity of fresh leaves. These conditions modulate genes involved in the biosynthesis of theanine, catechins, and aroma compounds, and increase the content of characteristic volatile aroma components. Optimal preservation efficacy is achieved within 2 to 6 h postharvest under such conditions [57].

Nevertheless, a standardized, data-driven technical system for fresh tea leaf storage and transportation has not yet been established in domestic industrial practice. The precise synergistic regulation of temperature and humidity, along with the optimization of dedicated ventilated storage and transportation equipment, represent core development directions for large-scale preservation technologies in the future.

### 4.2. Preservation Technologies for Finished Tea Products

#### 4.2.1. Drying and Humidity Regulation Preservation Technology

Moisture represents the core factor driving tea quality deterioration, and drying coupled with humidity regulation constitutes a fundamental prerequisite for all preservation technologies. Maintaining the moisture content of dried tea below 6% is a prerequisite for ensuring the efficacy of diverse preservation strategies. Traditional tea preservation practices predominantly employ desiccants including lime, charcoal, and silica gel for humidity management. These approaches offer operational simplicity and low cost but exhibit limited preservation precision. They are only suitable for short-term, small-scale storage and fail to accommodate the requirements of modern large-scale, standardized, long-term tea storage and distribution operations.

Optimal humidity conditions vary considerably across distinct tea categories. During dark tea storage, ambient relative humidity directly modulates the metabolite profiles and microbial community structure of raw dark tea. Appropriate humidity conditions maintain the stability of dark tea metabolites and preserve sensory quality during storage [8,58]. The shelf quality of flat-shaped green tea is co-determined by dried tea moisture content, storage temperature, ambient humidity, and packaging oxygen concentration. The synergistic combination of 4.5% dried tea moisture, 25 °C storage temperature, 55% relative humidity, and 0.2% packaging oxygen content maximally preserves the flavor and nutritional components of green tea [59].

#### 4.2.2. Low-Temperature Refrigeration Preservation Technology

Low-temperature refrigeration represents the most mature and widely applied physical preservation technology at present. Its core mechanism involves reducing the rate of physical and chemical reactions in tea, inactivating endogenous enzyme activity, and suppressing microbial metabolism via low-temperature exposure. This approach effectively delays tea aging and deterioration processes, including oxidative browning and aroma attenuation. The aging rate of tea exhibits a positive correlation with ambient temperature, and low-temperature conditions effectively preserve core flavor constituents, including tea polyphenols, amino acids, and aroma compounds.

Currently, the mainstream preservation temperature adopted in the industry ranges from 0 °C to 5 °C. This temperature range avoids damage to tea cellular structures and maximally preserves the inherent quality of tea products. Storage temperatures below 8 °C significantly decelerate amino acid degradation. For highly sensitive tea categories such as premium green tea, refrigeration below 5 °C and ultra-low-temperature storage below −10 °C enable long-term preservation. Optimal low-temperature conditions vary across different application scenarios. Temperatures around 8 °C are suitable for long-distance cold-chain transportation of fresh leaves. This temperature range stabilizes characteristic metabolites including catechins and purine alkaloids via modulating key genes and transcription factors, thereby reducing processing losses at the raw material stage [2]. A temperature of 15 °C is appropriate for short-term withering and temporary storage of fresh leaves. When combined with enclosed CO_2_ regulation, this condition further enhances chlorophyll protection efficacy [16]. Furthermore, low-temperature environments stabilize glycosidic aroma precursors in green tea, exerting a beneficial effect on aroma retention [60].

Divergent perspectives exist in the academic community regarding the optimal storage temperature for green tea. Dai et al. [61] reported that ultra-low temperatures of −80 °C and −20 °C achieve the best retention of physicochemical components, chlorophyll, and volatile aroma compounds in green tea. By contrast, refrigeration at 4 °C and ambient-temperature storage accelerate endogenous substance degradation and aroma compound variation in green tea [61]. Conversely, Zhu et al. [43] proposed that refrigeration at 4 °C represents the optimal temperature for long-term green tea storage when preservation efficacy and operational costs are considered comprehensively. While freezing at −20 °C delivers outstanding preservation performance, it entails higher energy consumption and operational costs [43]. The discrepancy between these two conclusions primarily arises from differences in experimental tea cultivars, evaluation dimensions, and storage duration.

Nevertheless, low-temperature storage also exhibits distinct limitations. Low-temperature environments favor the survival of *Salmonella* in dried tea, elevating the risk of microbial contamination [62]. Additionally, cold storage is associated with high energy consumption, the increased susceptibility of tea to exogenous odor adsorption, and surface condensation and moisture absorption induced by temperature differentials during product removal. Accordingly, low-temperature refrigeration must be implemented in conjunction with hermetic odor-resistant packaging and gradual temperature acclimation procedures. From an industrial application perspective, low-temperature storage extends the shelf life of premium green tea and oolong tea by 6 to 12 months, representing the most mature technology with established cold-chain infrastructure, though its high energy cost and condensation-management requirements limit adoption for mid- and low-value teas and for small-scale operators without dedicated facilities.

#### 4.2.3. Modified Atmosphere and Deoxygenation Preservation Technology

Oxygen represents an essential prerequisite for oxidative deterioration of tea. Low-oxygen and anaerobic environments fundamentally suppress both enzymatic and non-enzymatic oxidation reactions in tea. Accordingly, modified atmosphere and deoxygenation preservation have emerged as mainstream high-precision preservation technologies for high-end tea products. Currently, mainstream industrial deoxygenation approaches fall into two primary categories, namely oxygen scavenger-mediated deoxygenation and vacuum nitrogen-flushing deoxygenation.

Iron-based oxygen scavengers are extensively applied in practice. Storage of Longjing tea at 5 °C and 25 °C for 18 months with oxygen scavengers effectively inhibits lipid and carotenoid oxidation and reduces stale flavor formation. The preservation efficacy under this approach is comparable to that of ultra-low-temperature storage at −20 °C [25]. Oxygen scavengers reduce the oxygen concentration within packaging to below 0.1%. They offer advantages including low cost, operational simplicity, no damage to tea morphology, and suitability for small-package distribution. Nevertheless, these materials release moisture during the oxygen absorption process, which readily induces elevation in tea moisture content. This adverse effect is more pronounced in small-package formats [27]. Vacuum deoxygenation represents another effective preservation strategy suitable for roasted green tea, achieving long-term oxygen isolation. When applied in combination with low-temperature and light-exclusion conditions, it maximally preserves the inherent aroma and taste profile of tea [35]. Deoxygenation packaging is suitable for long-term storage of Longjing tea, effectively inhibiting flavor deterioration and tea infusion yellowing. When combined with an appropriate dosage of tea oil, this approach further enhances preservation efficacy [24]. Under ambient-temperature storage conditions, the combined process of vacuum packaging and oxygen scavenger application delivers excellent color and aroma protection performance, with the overall quality comparable to that of low-temperature frozen storage [33].

Vacuum nitrogen-flushing preservation maintains the oxygen concentration within packaging at approximately 1%. By filling high-purity nitrogen to create an inert preservation environment, this technology delivers outstanding aroma and color protection efficacy and is suitable for the storage of high-end, high-aroma tea categories [44]. Accelerated shelf-life testing enables accurate calculation of the actual shelf life of small nitrogen-flushed packaged tea under varying temperature conditions [44]. Nevertheless, this technology exhibits distinct limitations. Once the package is opened, the inert environment is disrupted, and quality deterioration still occurs during subsequent storage, resulting in poor sustained efficacy. Standalone vacuum nitrogen flushing is susceptible to reduced preservation efficacy caused by nitrogen impurity and entrained water vapor, imposing considerable application constraints. Furthermore, optimal preservation technologies vary across tea categories. Domestic investigations have verified that oxygen extraction and nitrogen flushing technology delivers the best preservation efficacy for black tea. Foreign studies, by contrast, indicate that nitrogen-flushing preservation exhibits better adaptability to sencha than oxygen scavenger-based preservation. These findings collectively demonstrate that category-specific adaptation represents a key direction for optimizing oxygen-controlled preservation technologies.

In addition, the composite strategy combining vacuum treatment with preservatives achieves preservation efficacy comparable to low-temperature storage, with superior aroma retention performance. This approach provides a new technical pathway for low-cost, high-quality tea preservation. Overall, modified atmosphere deoxygenation preservation offers green, residue-free performance and strong aroma protection capacity. However, it imposes extremely high requirements for packaging sealing integrity and equipment precision and entails high production costs. Currently, large-scale widespread application in bulk tea storage remains challenging, and the technology is economically scalable primarily for small-format high-value tea packaging rather than bulk warehouse storage.

#### 4.2.4. Preservative-Assisted Preservation Technology

Preservative-based preservation represents a precision auxiliary preservation technology developed on the basis of drying processes. It relies on food-grade additives to achieve multiple functions including dehumidification, deoxygenation, antioxidation, and bacteriostasis, thereby compensating for the limitations of standalone drying technologies. Existing preservatives exhibit limited long-term efficacy. Their activity declines gradually after three months of application, necessitating regular replacement. Furthermore, formulation composition and application mode directly determine application efficacy. Sodium metabisulfite and sodium ascorbate are typically formulated into composite desiccant sachets placed in the packaging headspace rather than in direct contact with tea leaves, where they release SO_2_ and exert antioxidant and deoxygenating effects. Trials demonstrate that such sachet-based composite systems, including sodium metabisulfite combined with sodium ascorbate and allochroic silica gel combined with sodium ascorbate, deliver superior preservation efficacy compared with conventional low-temperature refrigeration. Pure ascorbic acid, by contrast, is typically applied as a fine powder in direct contact with tea surfaces, which readily induces sour taste in tea infusions and presents application drawbacks [63].

Currently, research and development of tea preservatives is advancing toward natural and multifunctional directions. Synthetic antioxidants including vitamin C, erythorbic acid, and sodium erythorbate are extensively applied, and research breakthroughs have also been achieved for green additives such as natural plant-derived preservatives and food-grade enzyme preparations. Furthermore, reduced glutathione, as a novel food-grade preservation additive, binds and protects volatile aroma compounds in tea and increases the content of active components including tea polyphenols and umami amino acids. It exhibits promising application prospects in the field of aroma protection and preservation of liquid tea products [12].

Overall, preservatives offer low cost and convenient application, but standalone preservatives cannot satisfy full-cycle preservation requirements. Additionally, certain chemical preservatives carry potential safety risks. The long-term efficacy of natural preservatives remains to be further optimized, and they are predominantly applied as auxiliary technologies in composite preservation systems.

#### 4.2.5. Irradiation Preservation Technology

Irradiation preservation represents an ambient-temperature cold sterilization preservation technology applied across grain, fruit, and vegetable systems [64], and its feasibility in tea has been demonstrated in several tea-specific studies. The application of ^60^Co γ-rays or high-energy electron beams achieves sterilization, bacteriostasis, and inactivation of residual enzyme activity without temperature elevation, effectively addressing mold and pest issues during tea storage. In black tea, γ-irradiation at 7 and 10 kGy maintained stable theaflavin, thearubigin, and liquor color parameters throughout 12 months of storage while achieving complete microbial decontamination and reducing moisture absorption from 8.65% to 6.0% under high-humidity conditions [65]. For black broken tea, the D_10_ value for total bacterial count was 1.70 kGy for ^60^Co γ-rays and 2.66 kGy for electron beams, with 6.5 kGy γ-irradiation reducing microbial load to below the detection limit [66]. This technology offers advantages including ambient-temperature operation, no chemical residue, and no impairment to the basic flavor profile at appropriate dosages, making it suitable for long-term storage and batch distribution of bulk tea products.

Existing investigations demonstrate that low-dose γ-rays achieve effective sterilization and bacteriostasis, whereas high doses induce irreversible damage to tea aroma and amino acid components, leading to altered aroma profiles and flavor deterioration [67]. Precise regulation of irradiation dosage represents the core challenge for technological breakthroughs in this field. Meanwhile, consumer acceptance of irradiated agricultural products remains low, and market recognition is limited. These factors substantially constrain industrial promotion and large-scale application of this technology. Currently, irradiation preservation technology is only applied as a niche auxiliary preservation approach and has not yet developed into a mainstream industrial preservation solution, with most published evidence limited to laboratory-scale dosimetry and microbial-load testing rather than long-term commercial storage trials.

#### 4.2.6. Packaging Material and Container Preservation Technology

Packaging materials and storage vessels serve as core barriers against external moisture, oxygen, light, and exogenous odors, and maintain the stability of the tea storage microenvironment. The barrier performance of constituent materials directly determines tea preservation efficacy [37].

Preservation capacity and tea polyphenol protection efficacy vary considerably across packaging materials of different compositions. Paper cartons and conventional polyethylene bags exhibit weak moisture and oxygen barrier capacity. Iron tins and conventional plastic composite bags deliver moderate performance. Aluminum foil composite bags demonstrate outstanding comprehensive barrier performance, effectively reducing tea moisture absorption and quality deterioration [33]. Conventional transparent polyethylene bags exhibit limited barrier capacity against oxygen, light, and moisture, leading to rapid loss of tea polyphenols. Hermetic metal cans deliver superior comprehensive barrier performance, effectively slowing tea polyphenol oxidation and extending the shelf life of green tea [13]. Under simulated deterioration conditions of 37 °C, 85% relative humidity, and 800 lx illumination, tinplate combined with aluminum foil composite bag packaging delivers the best preservation efficacy for Xinyang Maojian tea, followed by standalone aluminum foil composite bags. Single tinplate cans and kraft paper bags exhibit the poorest performance. Composite packaging restricts the increase in tea moisture content to within 1.55%, with a sensory attenuation rate of only 26% [68].

Trials conducted on Biluochun tea further corroborate that tinplate cans and multi-layer composite films deliver superior preservation efficacy compared with paper packaging under ambient-temperature conditions [69]. For Longjing tea stored in high-temperature environments, metal cans exhibit rapid heat conduction and tend to accelerate aroma deterioration, whereas heat-insulating composite paper packaging offers more favorable performance [70]. Polyethylene, PET, and aluminum–plastic composite films exhibit low adsorption affinity for green tea aroma compounds and low propensity for impurity migration, demonstrating high application suitability. Polypropylene materials, by contrast, may release off-flavor compounds and readily induce flavor deterioration [46]. Nano-modified packaging delivers comprehensively enhanced barrier and mechanical properties, with substantially higher retention rates of tea active components compared with conventional packaging. Under both ambient and high-temperature storage conditions, it effectively delays the oxidative decomposition of endogenous substances in green tea and better preserves the inherent color, aroma, and taste profile of tea after long-term storage [71].

Storage vessels exert a pronounced influence on the flavor profile of post-fermented teas. Relative to original factory hermetic packaging, storage of ripe Pu-erh tea in pottery jars, iron tins, wooden boxes, and cartons enhances infusion sweetness and intensifies aged aroma notes. 1,2,4-Trimethoxybenzene has been identified as a characteristic marker compound for differentiating flavor profiles induced by different storage vessels [72]. Black tea exhibits a short-term aroma enrichment effect during storage, with sensory scores following an initial increase followed by a subsequent decline. High-barrier composite packaging delays the arrival of the aroma peak and extends the maintenance period of tea aroma quality. The core mechanism underlying this effect involves reduction in the dissipation rate of aroma compounds [47]. Meanwhile, metal and original factory packaging delay chlorophyll degradation in green tea, whereas glass and paper packaging accelerate pigment loss under light exposure [17]. Furthermore, green tea packaging selection requires comprehensive matching with storage temperature scenarios and cannot rely solely on material performance grade.

Currently, tea packaging is gradually upgrading toward multi-layer composite, nano-modified, degradable, and intelligent functional designs. Functional packaging can be flexibly combined with low-temperature and modified atmosphere technologies, representing a supporting preservation approach with relatively high industrial implementability for standard product formats, though intelligent and nano-modified variants remain at early commercialization stages with limited cost data.

### 4.3. Biological Preservation and Composite Synergistic Preservation Technologies

Biological preservation represents an environmentally friendly technology with advantages including green safety, low energy consumption, and no impairment of tea appearance and flavor. It encompasses two technical pathways. Microbial film-forming preservation, in which low-temperature-adapted Bacillus powder forms a protective film under low-oxygen packaging to block oxygen contact, has been explored in fresh-cut produce systems [73], and preliminary attempts in tea have been reported with limited efficacy [74]. Its reliable application in tea remains to be validated. Enzyme activity inactivation reduces residual oxidase activity in oolong tea and green tea via high-temperature short-time re-drying and microwave treatment. Tea-specific studies have verified that microwave enzyme inactivation significantly improves the storage stability and comprehensive quality of green tea, attenuating oxidative deterioration at the source. The enzyme inactivation approach is applicable under ambient-temperature conditions, while the microbial film-forming pathway requires further systematic investigation before its efficacy in tea matrices can be confirmed.

In addition to biological preservation technologies, novel preservation approaches including ultrasonic pretreatment, ultra-high pressure preservation, microcapsule-based aroma protection, and microencapsulation continue to evolve. These technologies address key pain points of tea quality deterioration during storage via respective mechanisms, including residual enzyme inactivation, free water removal, ambient-temperature sterilization and color protection, and volatile aroma compound encapsulation, thereby compensating for the limitations of traditional technologies. Among these approaches, microcapsule technology delivers outstanding performance for deeply processed tea products such as instant tea. It employs wall materials to encapsulate aroma and heat-sensitive compounds, reducing losses induced by high-temperature storage. Experimental evidence demonstrates that gelatin achieves the highest embedding efficiency as a wall material, with comprehensive performance superior to that of maltodextrin, gum acacia, and other alternative materials [26]. Table 3 presents a comprehensive comparison of mainstream tea preservation technologies with respect to working principle, strengths, limitations and industrial application maturity. Most preservation strategies have only been validated under constant laboratory conditions, and their performance in real fluctuating circulation environments remains to be verified.

It should be noted that all standalone preservation technologies exhibit distinct inherent limitations. Low-temperature preservation entails high energy consumption and increased susceptibility to exogenous odor adsorption. Preservatives exhibit limited long-term efficacy. Modified atmosphere preservation requires high equipment investment and imposes stringent sealing requirements. Standalone biological preservation delivers insufficient stability. No single technology can simultaneously satisfy diverse industrial requirements for color protection, aroma preservation, bacteriostasis, long-term storage, low cost, and easy promotion. Composite preservation modes featuring multi-technology coupling have emerged as an inevitable development trend. Mainstream industrial combinations include drying pretreatment coupled with low-temperature refrigeration and high-barrier packaging, nitrogen-flushed modified atmosphere combined with natural biological preservatives and full light-exclusion storage, and microwave enzyme inactivation integrated with nano-packaging and low-temperature storage and transportation. These strategies achieve all-dimensional preservation via complementary advantages of multiple technologies. The combination of oxygen scavengers and desiccants also addresses the moisture release issue associated with oxygen scavengers, representing a high-quality composite strategy for premium green tea [27].

Currently, the majority of novel preservation technologies and composite processes remain in the laboratory research stage. They are characterized by non-standard process parameters, high equipment transformation costs, and insufficient large-scale adaptability, and their performance under real fluctuating circulation conditions rather than constant laboratory environments remains to be verified. Future research efforts should focus on further process optimization, cost reduction, technical standard unification, and industrial technology implementation. Meanwhile, integration with the Internet of Things and intelligent sensing technologies will enable the development of intelligent warehousing systems, advancing the field toward green, precise, and intelligent development directions.

Overall, mainstream tea preservation technologies differ markedly in functional positioning, applicable scenarios and industrial maturity, presenting a clear echelon distribution pattern. In terms of core preservation efficacy, low-temperature refrigeration and modified atmosphere deoxygenation constitute the first echelon with comprehensive quality retention performance under controlled conditions. Drying humidity regulation and packaging barrier technologies serve as indispensable basic supporting measures, while irradiation and biological preservation only play auxiliary roles due to their limited protective effect on trace aroma components. From the perspective of industrial adaptability, low-temperature storage and high-barrier composite packaging offer the highest technical maturity and the most complete industrial supporting systems among current options, but their high energy consumption and operating costs restrict popularization in mid- and low-end tea products. Oxygen scavenger technology delivers the optimal cost-performance for small-packaged products, while novel approaches such as biological preservation and intelligent packaging remain at the laboratory pilot stage with poor large-scale applicability.

Notably, a prominent mismatch exists between current research hotspots and actual industrial demands. Most laboratory studies prioritize highly innovative but poorly implementable novel technologies, while core industrial pain points including bulk tea cold storage parameter standardization, large warehouse humidity control and aroma retention during circulation lack sufficient targeted research. The gap between laboratory efficacy and industrial scalability remains a primary bottleneck, and technology transfer requires sustained pilot-scale validation before widespread adoption can be recommended. Meanwhile, the over-reliance of most studies on routine component indicators such as polyphenols and amino acids, rather than systematic aroma retention and sensory quality evaluation, also largely limits the industrial translation of laboratory technical solutions.

## 5. Existing Problems and Challenges in the Field of Tea Preservation

Despite the establishment of a diversified preservation technology framework for tea in China, practical industrial implementation remains constrained by notable limitations that impede adaptation to the full-chain, high-quality storage and distribution requirements of tea products.

### 5.1. Absence of Category-Specific and Precision-Oriented Preservation Frameworks

Owing to variations in processing protocols and endogenous constituent profiles, the six major tea categories exhibit pronounced differences in storage deterioration patterns and preservation requirements. Roasted oolong teas, exemplified by Wuyi rock tea, are more amenable to ambient-temperature storage, which simultaneously optimizes flavor development and reduces cold storage operational costs. Nevertheless, prevailing preservation technologies predominantly rely on one-size-fits-all protocols, with limited development of tailored procedures and parameters customized for distinct tea categories and tea plant cultivars. Currently available cold-chain temperature control parameters for fresh leaves have only undergone molecular mechanism validation in the Yinghong No. 9 cultivar, while complementary storage and transportation datasets for diverse tea plant cultivars remain incomplete [2]. Furthermore, research efforts focused on the storage and preservation of specialty tea categories such as yellow tea remain generally underdeveloped. The root cause of this problem lies in the lack of a unified classification evaluation system for tea storage tolerance. Most studies carry out process optimization based on a single tea cultivar, and the conclusions lack cross-category universality, which makes it impossible to form a graded preservation parameter system covering all tea categories.

### 5.2. Notable Deficiencies in Aroma Preservation and Retention Technologies

Volatile aroma compounds in tea exhibit inherently limited chemical stability. While established preservation technologies effectively inhibit browning, mold spoilage, and taste deterioration in tea, they generally fail to achieve long-term retention of trace-level characteristic aroma components. Low-temperature conditions only exert protective effects on lipid-derived aroma precursors and are unable to achieve targeted stabilization of volatile aroma compounds. Inadequate aroma retention remains a core bottleneck restricting the long-term preservation of high-end premium tea products [2]. Current aroma preservation technologies mostly rely on passive barrier protection, lacking targeted regulatory strategies for aroma substance transformation pathways. Research on the interaction mechanism between packaging materials and aroma compounds, as well as molecular embedding targeted aroma stabilization technology, is still in its infancy and is the core direction for breakthrough in high-end tea preservation technology in the future.

### 5.3. Limited Industrial Translation and Standardization of Technological Systems

Cutting-edge approaches including composite preservation and intelligent preservation systems remain largely confined to the laboratory stage. Their high equipment costs and complex operational requirements hinder adaptation to large-scale production of bulk tea commodities. Furthermore, the industry lacks a standardized management framework covering storage, preservation, and storage–transportation segments.

Key operational indicators, including temperature and humidity setpoints, gas composition ratios, preservative dosages, and packaging specifications, lack industry-wide harmonization, which contributes to inconsistent preservation efficacy across market products. No unified industry standard exists for temperature control during fresh-leaf cold-chain transportation. The proposed optimal storage and transportation temperature of 8 °C has only been validated via small-scale trials and has not achieved widespread industrial adoption [2].

### 5.4. Delayed Deployment of Green Preservation Technologies

Certain traditional preservation modalities rely on chemical additives and high-energy storage infrastructure, which carry potential food safety risks and are misaligned with low-carbon development principles. Research, development, and the industrial translation of eco-friendly technologies, including natural plant-derived preservatives and degradable packaging materials, progress at a slow pace. A safe, efficient, and environmentally sustainable modern tea preservation system has yet to be fully established.

## 6. Conclusions and Prospects

The storage-induced quality deterioration of tea is driven by oxidative degradation of core bioactive components including polyphenols, amino acids, chlorophyll, and aroma compounds, with ascorbic acid and chlorophyll serving as reliable freshness indicators for green tea. Deterioration characteristics and preservation requirements differ significantly across tea categories and between fresh leaves and processed tea.

Building on these findings, this review proposes a whole-chain staged precise preservation framework with three differentiated regulatory objectives. At the fresh-leaf source end, low-temperature high-humidity cold-chain transportation maintains physiological activity and reduces mechanical damage. At the processing and storage end, a composite system of drying pretreatment, low-temperature or modified atmosphere treatment, and high-barrier packaging inhibits oxidative deterioration and maintains flavor stability according to category characteristics. At the terminal circulation end, functional packaging and small-batch sealed packaging delay quality attenuation in the open state. Unlike previously reported integrated preservation strategies focused primarily on the processed-tea storage stage, this framework extends to fresh-leaf cold-chain transportation, assigning stage-specific objectives and technologies and addressing open-state terminal distribution; this framework transcends the single-stage storage paradigm and replaces one-technology-fits-all with link-specific matching across source, process, and terminal links.

**Figure 2:**
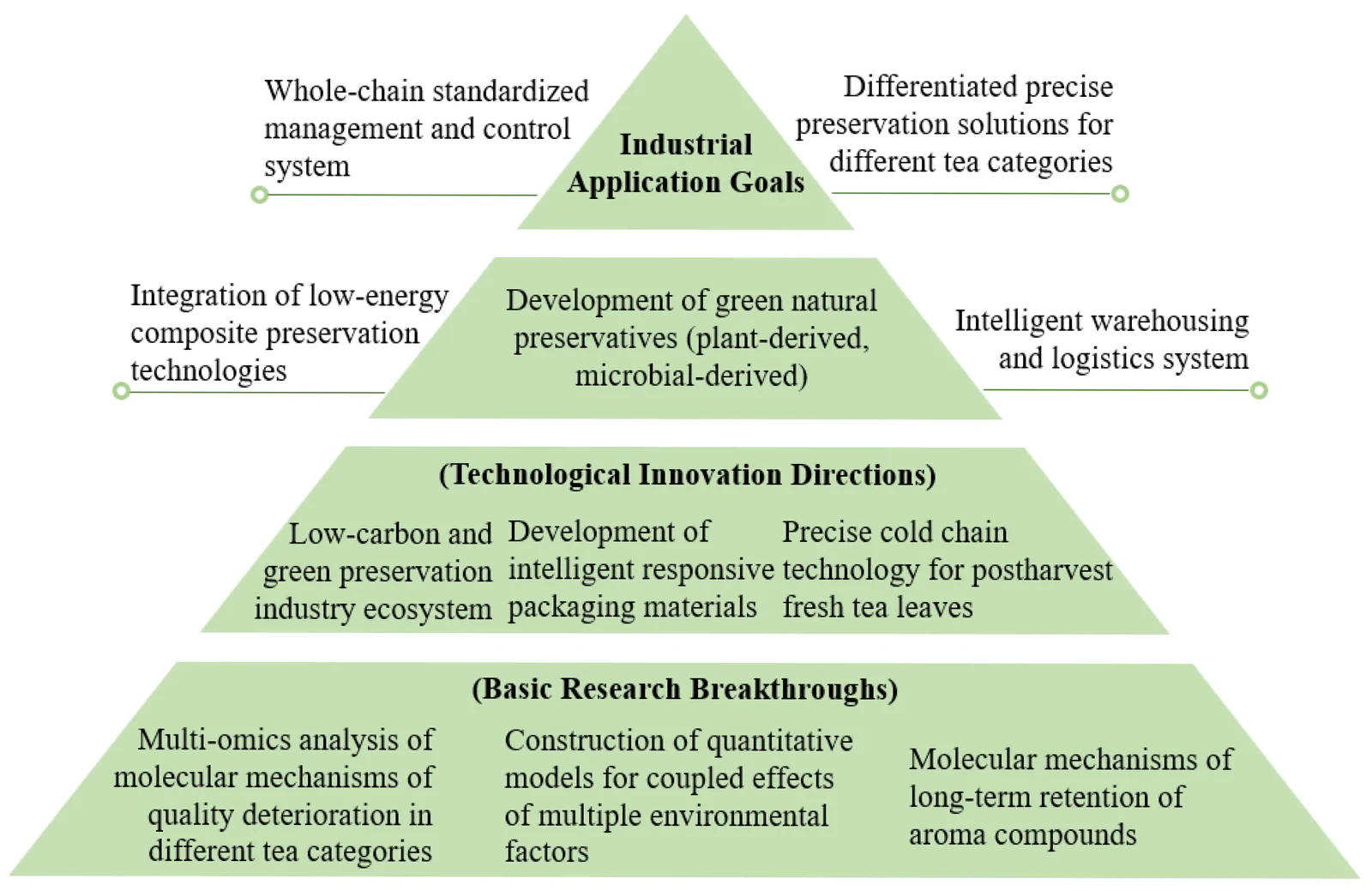
Future development directions and breakthrough paths of tea preservation technologies.

Key challenges remain in category-specific preservation systems, aroma retention technologies, industrial standardization, and green technology deployment, while yellow tea storage, deep-processed tea preservation, and harmful-substance evolution during storage remain underexplored. Roasting treatment has been demonstrated to alleviate stale flavor formation in stored green tea [28], and the overall development trend is outlined in Figure 2. Future efforts should prioritize targeted aroma stabilization, unified industry standards, and green intelligent facilities, alongside strengthened industry–university–research collaboration to reduce circulation losses and support the high-quality development of the tea industry.

## Figures and Tables

**Table 1:** Postharvest deterioration characteristics of fresh tea leaves and six major tea categories.

Tea Category	Core Quality Characteristics	Main Deterioration Manifestations	Dominant Deterioration Mechanisms	Most Sensitive Environmental Factors	Shelf Life Under Ambient Temperature Without Preservation	Characteristic Deterioration Markers	Reference
Fresh tea leaves	Bright green glossy appearance, soft texture, high endogenous enzyme activity	Water loss and wilting, increased leaf brittleness, reddening/browning from pile core overheating, mechanical injury, exogenous odor adsorption, rapid degradation of functional components	Sustained postharvest active respiration and transpiration, with accumulated field heat and respiratory heat; intensive enzymatic oxidation triggered by mechanical-injury-activated endogenous enzymes (PPO, POD); microbial proliferation under high-humidity conditions	Temperature, mechanical vibration, relative humidity	4–24 h (marked quality decline after 12 h, unsuitable for premium tea processing)	Browning index, chlorophyll fluorescence parameter Fv/Fm, branched-chain amino acids (valine, isoleucine), chlorophyll degradation rate	Chen et al. [2]
Dark tea	Aged and mellow aroma, thick red infusion	Mold proliferation, musty storage off-odor, harsh taste	Abnormal microbial metabolism, excessive polyphenol polymerization	Humidity, oxygen	Over 10 years (requires strict temperature and humidity control)	Aflatoxin (safety indicator under improper storage)	Lv, [8]
White tea	Pekoe fragrance with honey odor, sweet and mellow taste	Infusion darkening, reduced freshness, stale flavor development	Catechin degradation, EPSFs formation, amino acid transformation	Humidity	3–5 years (quality improves under proper storage)	EPSFs, pyroglutamic acid	Xie et al., [9]
Black tea	Red leaves and bright red infusion, sweet, mellow and brisk taste	Darkened leaf base, dull infusion, reduced briskness, rancid off-flavor	Oxidation polymerization of theaflavins and thearubigins into theabrownins, lipid oxidation rancidification	Moisture, oxygen	6–12 months	Theabrownins, linolenic acid	Cui, [10]
Green tea	Green leaves and clear infusion, fresh mellow taste with sweet aftertaste	Leaf yellowing, infusion darkening, aroma attenuation, taste flattening	Chlorophyll demagnesiumation and degradation; catechin oxidation and polymerization	Temperature, light	3–6 months	Chlorophyll a, EGCG, ascorbic acid	Hua et al. [11]
Oolong tea	Rich floral aroma; mellow taste with sweet aftertaste	Aroma loss, diminished taste intensity, fading roasted note	Oxidation of volatile terpenoids, slow polyphenol transformation	Oxygen, light	12–18 months	Hexanal, 2-methylbutanal	Xu et al. [12]
Yellow tea	Yellow leaves and infusion; sweet, mellow and fresh taste	Darkened leaf color, aroma loss, flat taste	Chlorophyll degradation, polyphenol oxidation	Temperature, light	6–12 months	Pheophytin	General guidance

PPO, polyphenol oxidase; POD, peroxidase. EPSFs, *N*-ethyl-2-pyrrolidinone-substituted flavan-3-ols. EGCG, epigallocatechin gallate.

**Table 2:** Reported storage environmental parameters and packaging specifications for different tea categories.

Tea Category	Reported Recommended Storage Temperature	Reported Recommended Relative Humidity	Maximum Safe Moisture Content (Dry Basis)	Recommended Oxygen Concentration	Recommended Packaging	Storage Precautions	Reference
Premium green teas	0–5 °C (refrigeration) or −18 °C (freezing)	45–55%	≤5.5%	≤0.1%	Aluminum foil composite bag + oxygen scavenger + tinplate can	35 °C hot-air tempering is required prior to removal from cold storage to prevent condensation	Li et al. [4]
Light-roasted oolong teas	0–5 °C	45–55%	≤5.5%	≤0.5%	Vacuum-sealed aluminum foil bag	Cool to ambient temperature after roasting prior to packaging	Zhang et al. [37]
Heavy-roasted oolong teas	15–25 °C (ambient storage)	50–60%	≤6.0%	≤5%	Pottery jar + sealed bag	Periodic re-roasting can extend shelf life	Huang et al. [38]
Young white teas (≤3 years)	0–5 °C (fir the first 3 years)	55–65%	≤6.5%	2–5%	Kraft paper bag + carton	May be transferred to ambient storage after 3 years of aging	General guidance
Aged white teas (>3 years)	15–25 °C	60–70%	≤7.0%	5–10%	Wooden box + cotton paper	Avoid frequent package opening	General guidance
Black teas	0-10 ℃	50-60%	≤6.0%	≤1%	Nitrogen-flushed aluminum foil bag	Avoid co-storage with odoriferous materials	Zhang, [49]
Dark tea (Pu-erh)	15–25 °C	55–65%	≤8.0%	5–15%	Bamboo basket + cotton paper wrapper	Maintain moderate ventilation, avoid fully airtight storage	Li et al. [50]

**Table 3:** Comparison of principles, advantages, disadvantages and industrial maturity of mainstream tea preservation technologies.

Technology Type	Core Principle	Main Advantages	Main Limitations	Core Application Scenarios	Reference
Low-temperature refrigeration	Slows biochemical reactions and inactivates endogenous enzyme activity	Broad applicability, reliable and well-established preservation efficacy	High energy consumption, prone to odor absorption and condensation upon removal from cold storage	Medium-to-long term storage of most tea categories, particularly temperature-sensitive premium green tea, black tea and high-aroma oolong tea	Li et al. [4]
Modified atmosphere and deoxygenation	Suppresses oxidative reactions by reducing ambient oxygen concentration	Excellent aroma and color retention, free of chemical residues	Strict requirements for packaging sealing, relatively high cost	Sealed storage of high-end premium and high-aroma teas, especially teas requiring strict oxygen isolation such as green tea, black tea and oolong tea	Wang et al. [25]
Drying and humidity control	Inhibits microbial growth by regulating moisture content	Low cost, simple operation	Only delays deterioration, cannot prevent aroma loss	Basic preservation support for all tea categories, suitable for high-humidity environments and simple household storage	Li et al. [33]
Preservative-assisted preservation	Exerts antioxidant, antibacterial and dehumidifying effects	Low cost, convenient application	Short effective duration, potential safety risks of some chemical agents	Auxiliary preservation for various teas, especially for ambient-temperature storage and long-distance transportation	Huang et al. [63]
Functional packaging and container preservation	Isolates external moisture, oxygen light and odors via high-barrier materials to stabilize the storage microenvironment	1. High adaptability as the core carrier of all preservation technologies 2. Multi-layer/nano-modified packaging delivers stable all-dimensional barrier performance 3. Customizable for diverse tea categories and storage scenarios	1. High cost of high-barrier materials, unsuitable for large-scale bulk tea 2. Ordinary plastic packaging tends to adsorb aroma and release off-flavors 3. Preservation efficacy is completely lost upon packaging damage	Full-cycle storage and circulation of all tea categories, fundamental support for tea preservation systems	Jiang et al. [68]
Irradiation preservation	Achieves cold sterilization and enzyme inactivation	Operates at ambient temperature, no chemical residues	Low consumer acceptance, high doses impair tea aroma	Batch sterilization of bulk and export teas, used only as a niche auxiliary method	Thomas et al. [65]
Biological preservation technology	Includes microbial film-forming and enzyme inactivation pathways; delivers protection via food-grade microorganisms and enzyme preparations	1. Green, safe and low-energy, aligned with low-carbon development 2. Preserves tea appearance and original flavor, suitable for ambient-temperature scenarios 3. Compatible with other technologies for comprehensive protection	1. Low technical maturity, mostly at laboratory research stage 2. Poor stability and long-term efficacy of biological agents 3. Strict environment requirements, poor adaptability to complex circulation	Ambient-temperature preservation of organic green tea and high-end premium teas, core development direction of future technologies	Liu et al. [73]; Chen [54]
Composite synergistic preservation	Achieves all-dimensional protection via multi-technology coupling (e.g., drying + cold storage + high-barrier packaging) through complementary advantages	1. Overcomes limitations of single technologies, with superior preservation efficacy 2. Flexibly customizable for different tea categories and storage scenarios 3. Balances efficacy, cost and safety, mainstream industrial trend	1. Complex process parameters, no unified industry standards 2. Higher production costs from combined technology application 3. Insufficient research on synergistic mechanisms, difficult process optimization	Full-chain quality control of high-end premium teas, cross-regional circulation teas and deep-processed products, current mainstream industry application mode	Zhu [27]

## Data Availability

No new data were created or analyzed in this study. Data sharing is not applicable to this article.
